# Numerical Study of Angle-Insensitive and Tunable Dual-Band THz Absorber Using Periodic Cross-Shaped Graphene Arrays

**DOI:** 10.3390/ma12132063

**Published:** 2019-06-27

**Authors:** Tian Sang, Jian Gao, La Wang, Honglong Qi, Xin Yin, Yueke Wang

**Affiliations:** 1 Department of Photoelectric Information Science and Engineering, School of Science, Jiangnan University, Wuxi 214122, China; 2 Jiangsu Provincial Research Center of Light Industrial Optoelectronic Engineering and Technology, Jiangnan University, Wuxi 214122, China

**Keywords:** graphene, THz absorber, angle-insensitive, tunable dual-band, Fabry-Pérot cavity resonance

## Abstract

A dual-band terahertz (THz) absorber using the periodic cross-shaped graphene arrays is presented. It is shown that the dual-band light absorption enhancement of graphene results from the edge graphene plasmon (EGP) resonance, and the locations of the two absorption peaks can be precisely estimated by using the Fabry-Pérot (F-P) cavity model. Slight residual reflection remains at the two absorption peaks because the input impedance of the cross-arm cannot be perfectly matched with the free space impedance. In addition, the locations of the two absorption bands can be simultaneously tuned by changing the Fermi level of graphene, and they can be independently tuned by changing the width or the length of the cross-arm of graphene. Excellent angle-insensitivity dual-band absorption enhancement of graphene can be maintained for both the transverse electric (TE) and transverse magnetic (TM) polarizations.

## 1. Introduction

In recent years, terahertz (THz) technology has become one of the most attractive research topics, because of its promising applications in the fields of imaging, spectroscopy, security and communications [1]. The THz absorber is an important branch of THz technology, which can find practical applications in the above fields [2,3]. In the past decade, metallic metamaterials and metasurfaces have been used to develop THz absorbers [4,5,6]. More recently, graphene has been demonstrated as a good complementary material in realizing THz absorbers, because it can support surface plasmon polariton (SPP) in THz and far-infrared regions [7,8]. The overlap between graphene physics and SPP, graphene plasmon (GP), opens up new approaches to enhance light-matter interaction due to the remarkable properties of graphene, such as high carrier mobility, electrical tunability and strong light confinement [9,10].

Various groups have been working on graphene-based absorbers in the THz region. Many of these absorbers are based on periodically patterned graphene, like disks [11,12], microrings [13], nanoribbons [14,15], elliptical arrays [16,17], cross-shaped [18,19], H-shaped [20] and dumbbell-shaped [21] structures. However, most of these graphene-based absorbers have only one absorption band, and their absorption efficiency is relatively low. To improve the light absorption efficiency, or to increase the number of the absorption bands of graphene, more complicated structures, such as multilayer structures [22,23,24,25] and cascaded graphene patterns [26,27,28,29] are proposed. However, these structures rely multilayer optical coatings or multileveled graphene patterns, which reliance results in additional challenges in fabrication. Undoubtedly, a graphene-based tunable dual-band THz absorber with a relatively simple structure and a high absorption efficiency is highly desired.

In this work, we numerically investigate a tunable dual-band THz absorber with a high absorption efficiency by using the periodic cross-shaped graphene arrays. The dual-band light absorption enhancement of graphene results from the edge graphene plasmon (EGP) resonance, and a simplified Fabry-Pérot (F-P) cavity model is proposed to estimate the locations of the two absorption peaks. The absorption bands of graphene can be simultaneously tuned by changing the Fermi level of graphene, and they can be independently tuned by changing the width or the length of the cross-arm of graphene. Angle-insensitivity dual-band absorption enhancement of graphene can be maintained for both the transverse electric (TE) and transverse magnetic (TM) polarizations. Besides the advantages of high absorption efficiency and relatively simple structure, the absorption performance of the proposed structure could be tuned more flexibly because it has one more free geometry parameter compared with other simple structures such as graphene disk and nanoribbon.

## 2. Structure and Model

**Figure 1:**
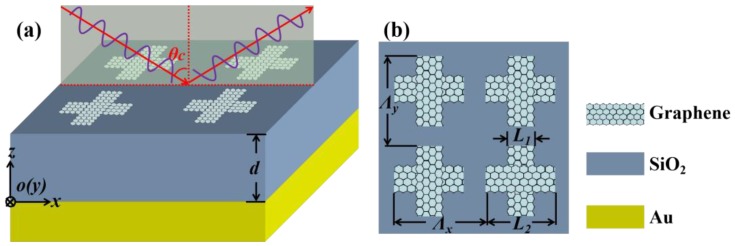
(**a**) Schematic diagram of the proposed absorber consisting of cross-shaped graphene arrays. From the top to the bottom are periodic cross-shaped graphene arrays, a thin SiO_2_ spacer with the thickness of *d*, and a semi-infinite Au substrate, respectively. (**b**) Vertical view of the cross-arm of graphene with period *Λ_x_* = *Λ_y_*, width *L*_1_ and length *L*_2_.

Figure 1 shows the schematic diagram of the proposed absorber illuminated by the TM plane wave (the magnetic-field vector lies along the *y*-axis). The absorber consists of the periodic cross-shaped graphene arrays with width *L*_1_, length *L*_2_ and period *Λ_x_* = *Λ_y_*. The cross-shaped graphene adheres to a metallic reflective plate substrate (Au film) separated by a thin silicon dioxide (SiO_2_) spacer with the thickness of *d*. Here, the reflective Au plate can be equivalent to the perfect electric conductor, which can efficiently enhance the interaction between the incident light and the patterned graphene arrays, due to the mirror effect. From the point of view of device fabrication, the Au film and thin SiO_2_ spacer can be deposited by using the conventional electron beam evaporation technique [30], and the patterned graphene of the cross-shaped arrays can be fabricated by using electron beam lithography and oxygen plasma etching [31]. The relative permittivity of SiO_2_ is *ε_d_* = 3.9 [18,32]. The surface conductivity of graphene *σ_g_* can be modeled by the Kubo formula including interband and intraband transitions [33,34,35]:

(1) $$\sigma_{g}=\frac{2ie^{2}k_{B}T}{\pi \hbar^{2}\left(\omega +i\tau^{-1}\right)}\ln\left[2\cosh\left(\frac{E_{f}}{2k_{B}T}\right)\right]+\frac{ie^{2}}{4\pi \hbar }\ln\left[\frac{2E_{f}-\left(\omega +i\tau^{-1}\right)\hbar }{2E_{f}+\left(\omega +i\tau^{-1}\right)\hbar }\right]$$

where *e* is the electron charge, *k*_B_ is the Boltzmann constant, *E_f_* is the Fermi level, *T* is the temperature, *ħ* is the reduced Planck constant, *τ* is the momentum relaxation time, and *ω* is the angular frequency of optical excitation. The relative permittivity of graphene *ε_g_* can be characterized as [36]:

(2) $$\varepsilon_{g}=1+\frac{i\sigma_{g}}{\varepsilon_{0}\omega t}$$

where *t* = 0.34 nm is the thickness of graphene layer and *ε*_0_ is the vacuum permittivity. According to Equations (1) and (2), the relative permittivity of graphene can be modulated because its Fermi level can be tuned by changing the chemical doping or applying bias voltage.

**Figure 2:**
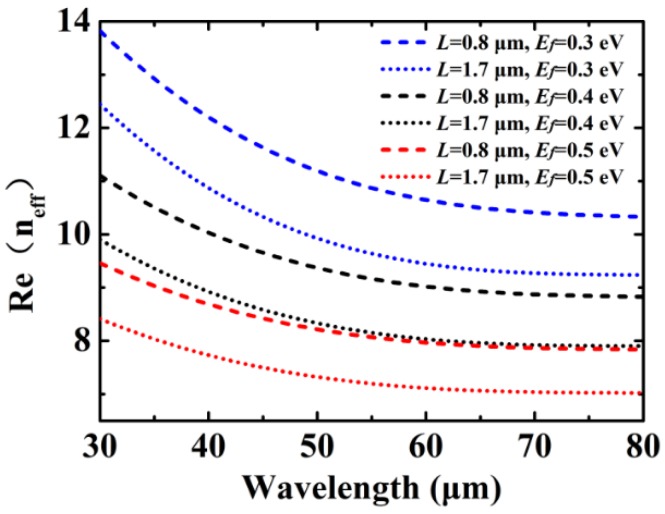
Real part of the effective refractive index Re(*n_eff_*) of the GP vs. wavelength under different widths *L* and Fermi levels *E_f_*.

Along the directions of the *x* or *y* axis, the cross-arm of graphene can be approximatively viewed as a finite-sized graphene nanoribbon. Based on Finite Element Method (FEM) simulation, we firstly perform a modes analysis of the graphene nanoribbon under different widths *L* and Fermi levels *E_f_*. Here, only the dispersion relation of the fundamental mode of the GP is analyzed. Figure 2 shows real part of the effective refractive index Re(*n_eff_*) of the GP as function of wavelength under different widths *L* and Fermi levels *E_f_* of the graphene nanoribbon. As can be seen in Figure 2, the propagation characteristics of the GP can be modulated by changing the Femi level *E_f_* and the width *L* of the graphene nanoribbon, which provides an effective strategy to control the resonant wavelengths of the GP modes.

## 3. Simulation Results and Analysis

**Figure 3:**
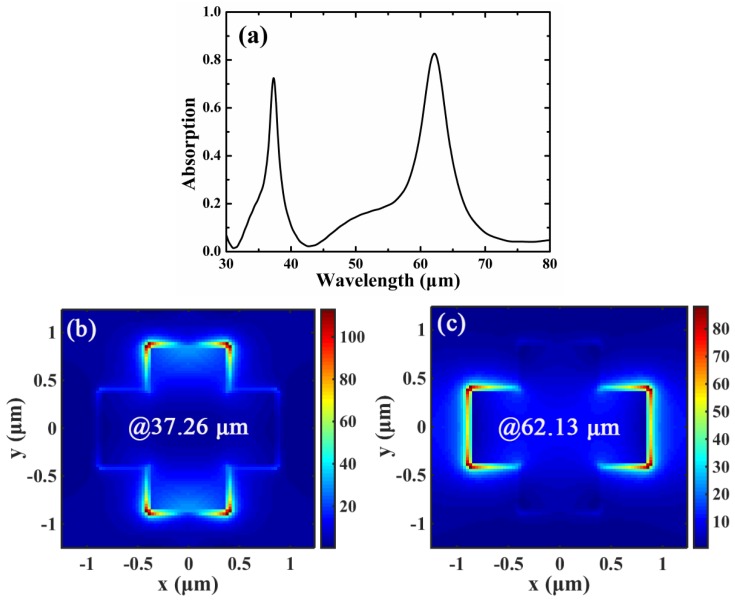
(**a**) Absorption response of the cross-shaped graphene arrays. (**b**) and (**c**) are the normalized electric-field distributions of the cross-arm in the *xy* plane for absorption peaks of 37.26 μm and 62.13 μm, respectively.

**Figure 4:**
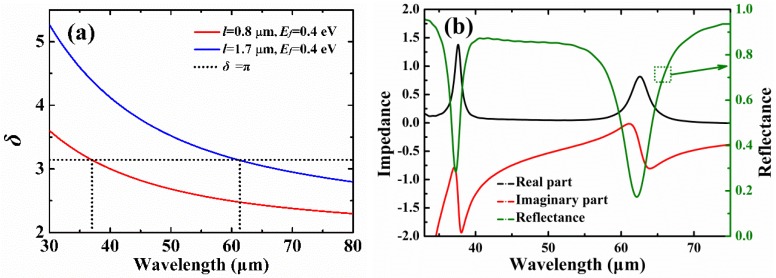
(**a**) Resonance wavelengths estimated by using the F-P cavity model for different cavity lengths with *E_f_* = 0.4 eV. (**b**) Reflection response and input impedance of the cross-shaped graphene arrays.

In simulation, the 3D finite-difference time-domain (FDTD) models of the commercial software FDTD Solutions are used to analyze the absorption properties of the proposed absorber. The absorption of graphene can be simplified as *A* = 1 − *R*, where *R* is reflection, which is because the transmission channel is blocked by the optically thick Au film. Figure 3a shows the absorption response of the structure shown in Figure 1. The parameters are: *Λ_x_* = *Λ_y_* = 2.5 μm, *E_f_* = 0.4 eV, *τ* = 0.5 ps, *d* = 4 μm, *T* = 300 K, *L*_1_ = 0.8 μm and *L*_2_ = 1.7 μm. The incident angle is *θ_c_* = 0, and the direction of the incident electric-field is along the *x*-axis. As shown in Figure 3a, two bands with high absorptivity occur at the wavelength of 37.26 μm and 62.13 μm, and the corresponding absorptions are 72.42% and 82.73%, respectively. The normalized electric-field distributions of the cross-arm in the *xy* plane for 37.26 μm and 62.13 μm are illustrated in Figure 3b,c. As can be seen in Figure 3b,c, the electric-field of the cross-arm is significantly enhanced for both of the two peak wavelengths. In addition, the enhanced electric-field tends to be concentrated on the edges of the cross-arm along the *x*-axis, indicating the features of the EGP modes [33]. In particular, for the absorption wavelength of 37.26 μm, the electric-field distribution of the cross-arm is highly confined on the edges of width *L*_1_; similarly, the electric-field is well confined on the edges of length *L*_2_ for 62.13 μm. Here, we consider the cross-arm as an F-P cavity with different cavity lengths along the *x*-axis (the direction of the incident electric-field), and an enhanced absorption peak can be realized when the cross-arm is in F-P resonance. The F-P cavity resonance condition can be described as [37]:

(3) δ=2πlRe(neff)λ+ϕ=mπ

where *δ* is the phase shift, *λ* is the resonance wavelength, *l* is the F-P cavity length of graphene, *ϕ* is the additional phase, and *m* is an integer which represents the resonance order. As shown in Figure 4a, the resonance wavelengths calculated by using the F-P cavity model for *l* = 0.8 μm and *l* = 1.7 μm with *E_f_* = 0.4 eV are 36.92 μm and 61.16 μm, respectively. The theoretical results of the F-P cavity model are in good agreement with the results of 37.26 μm and 62.13 μm calculated by using the FDTD method.

To better understand the selective absorption enhancement of the cross-shaped graphene arrays, the input impedance of the structure are studied by using the impedance theory. According to the impedance theory [38,39], the relation between the *S* parameters and impedance *Z* can be expressed as:

(4) $$S_{21}=S_{12}=\frac{1}{\cos(nkd)-\frac{i}{2}\left(Z+\frac{1}{2}\right)\sin(nkd)}$$

(5) $$S_{11}=S_{22}=\frac{i}{2}\left(\frac{1}{Z}-Z\right)\sin(nkd)$$

where *S*_11_, *S*_21_, *S*_12_, *S*_22_ are *S* parameters, *n* is the effective refractive index of the structure, and *k* the wave vector. The input impedance *Z* of the structure can be written as [40]:

(6) $$Z=\pm \sqrt{\frac{{\left(1+S_{11}\right)}^{2}-S_{21}^{2}}{{\left(1-S_{11}\right)}^{2}-S_{21}^{2}}}$$

therefore, the reflection of the cross-shaped graphene arrays can be calculated as $R={\left(\frac{Z-Z_{0}}{Z+Z_{0}}\right)}^{2}$, where *Z*_0_ is the impedance of the free space, and $Z_{0}=\sqrt{\mu \left(\omega \right)/\varepsilon \left(\omega \right)}=1$. Obviously, the impedance of the structure should be well matched with that of the free space, so as to reduce reflection. Figure 4b shows reflection response and the input impedance of the absorber, and parameters are the same as those in Figure 3a. As can be seen in Figure 4b, both the real and imaginary parts of *Z* are varied abruptly around the two absorption bands, but slight residual reflection remains at the locations of the resonance wavelengths because the input impedance cannot be perfectly matched with the free space impedance.

**Figure 5:**
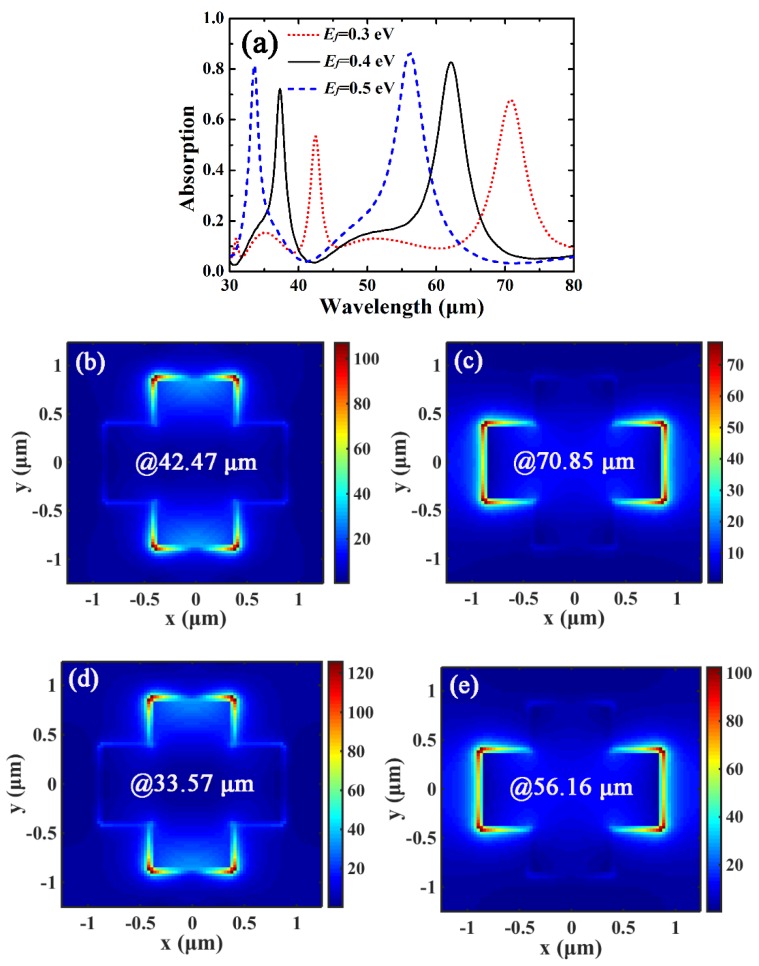
(**a**) Absorption spectra of the cross-shaped graphene arrays for different *E_f_*. (**b**,**c**) are the normalized electric-field distributions of the cross-arm with *E_f_* = 0.3 eV in the *xy* plane for absorption peaks of 42.47 μm and 70.85 μm, respectively. (**d**,**e**) are the normalized electric-field distributions of the cross-arm with *E_f_* = 0.5 eV in the *xy* plane for absorption peaks of 33.57 μm and 56.16 μm, respectively.

Figure 5 shows the absorption properties of the cross-shaped graphene arrays under the influence of the Fermi level, while the other parameters are the same as those in Figure 3a. As can be seen in Figure 5a, the locations of the two absorption peaks can be simultaneously tuned by changing the Fermi level *E_f_*. The locations of the two absorption peaks are blue-shifted with the increase of *E_f_*. As *E_f_* is increased from 0.3 eV to 0.5 eV, the absorption peak at the shorter wavelength is decreased from 42.47 μm to 33.57 μm, and the absorption peak at the longer wavelength is decreased from 70.85 μm to 56.16 μm as well. The blue-shift of the absorption peaks can be explained by the F-P cavity theory. As shown in Figure 2, the real part of the effective refractive index Re(*n_eff_*) of the GP is decreased as *E_f_* is increased, thus the locations of the absorption peaks are blue-shifted with the increase of *E_f_* according to Equation (3). In addition, the peak absorption of the two bands is slightly increased as *E_f_* is increased. This is because the surface conductivity of graphene *σ_g_* is increased with the increase of *E_f_*, thus the light absorption of graphene at the peak wavelength can be further improved by increasing *E_f_*. As shown in Figure 5b–e, the amplitude of the normalized electric-field of the cross-arm with *E_f_* = 0.3 eV is smaller than that of with *E_f_* = 0.5 eV, and stronger EGP resonance can be realized due to the increase of charge oscillations as *E_f_* is increased, resulting in enhanced light absorption of graphene for both of the peaks.

**Figure 6:**
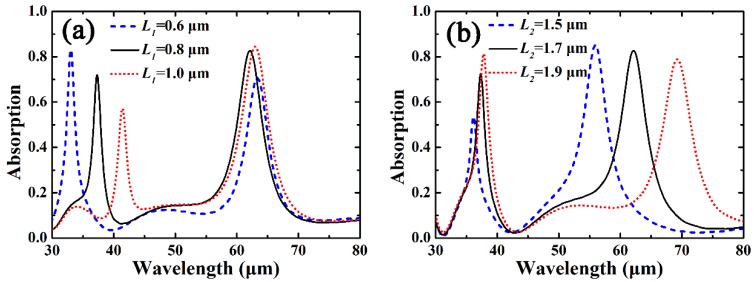
(**a**) Influence of width *L*_1_ on the absorption performance of the cross-shaped graphene arrays. (**b**) Influence of length *L*_2_ on the absorption performance of the cross-shaped graphene arrays.

Figure 6 shows influences of width *L*_1_ and length *L*_2_ on the absorption performance of the cross-shaped graphene arrays, and other parameters are the same as those in Figure 3a. As can be seen in Figure 6a, the location of the absorption peak at the shorter wavelength is red-shifted as *L*_1_ is increased, while the location of the absorption peak at the longer wavelength is almost immune to the variation of *L*_1_. This is because the absorption peak at the shorter wavelength is excited by the F-P cavity resonance of *L*_1_, thus its location will be redshifted as *L*_1_ is increased. Similarly, as shown in Figure 6b, because the absorption peak at the longer wavelength is excited by the F-P cavity resonance of *L*_2_, the corresponding location of the absorption peak can be effectively tuned by varying *L*_2_. Therefore, the locations of the two absorption peaks can be independently tuned by changing the width or the length of the cross-arm. 

Additionally, because the amplitude of the normalized electric-field of the cross-arm is changed as *L*_1_ or *L*_2_ is altered, the variation of *L*_1_ or *L*_2_ will influence the intensity of the two absorption peaks. Note because the absorption bandwidth at the shorter wavelength is narrower, thus its intensity at the absorption peak is more sensitive to the variation of *L*_1_ or *L*_2_.

**Figure 7:**
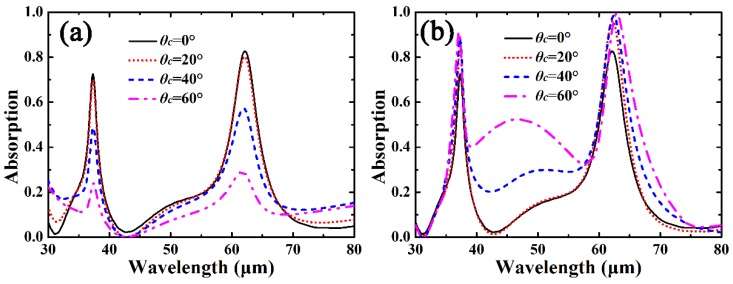
Absorption response of the cross-shaped graphene arrays as a function of the incidence angle for (**a**) TE and (**b**) TM polarizations. Other parameters are the same as those in Figure 3a.

Finally, we investigate the angular robustness of the proposed graphene-based dual-band THz absorber. Figure 7 shows absorption response of graphene as a function of the incidence angle for the TE (electric-field vector lies along the *y*-axis) and TM polarizations, whereas other parameters are the same as those in Figure 3a. As can be seen in Figure 7, the absorption response of graphene is robust to the variation of the incident angle for both the TE and TM polarizations. As shown in Figure 7a, as *θ_c_* is increased from 0° to 40°, the peak absorption of the two absorption bands is still larger than 48.8%, which is comparable to many graphene-based THz selective absorbers [12,13,14,15,16,17,18,19,20,21,29]. In particular, as shown in Figure 7b, the peak absorption of the absorption bands for the TM polarization can be further improved as *θ_c_* is increased, and perfect light absorption of graphene with *A* = 99.4% can be achieved as *θ_c_* = 60°. The increase of the light absorption of graphene at the peak wavelength may result from stronger confined electric-fields at oblique incidence for the TM polarization [18]. In addtion, because the direction of the magnetic field for the TM polarization remains constant as *θ_c_* is altered, the strength of magnetic resonance can be sufficiently kept and further ensures the high ohmic loss in graphene, thus the absorption response of the TM polarization is more robust to the variation of the incident angle [41]. Note the variation of incident angle *θ_c_* almost does not affect the F-P cavity length of the cross-arm, thus the locations of the two absorption peaks are kept almost the same even if *θ_c_* is significantly altered for both the TE and TM polarizations.

## 4. Conclusions

In conclusion, we numerically study an angle-insensitive and tunable dual-band THz absorber consisting of periodically patterned cross-shaped graphene arrays. The enhanced light absorption of graphene originates from the EGP resonance, and the locations of the two absorption peaks can be estimated by using the simplified F-P model. Excellent agreements have been achieved between the results of the FDTD and the F-P cavity model. The impedance theory is used to evaluate the absorption properties of graphene, which shows that slight residual reflection remains at the absorption peaks because the input impedance cannot be perfectly matched with that of the free space. In addition, the absorber shows flexible adjustability and excellent angle-insensitive absorption performances. The locations of the dual-band absorption can be simultaneously tuned by changing the Fermi level of graphene, and they can be independently tuned by changing the width or length of the cross-arm of graphene. An excellent angle-insensitivity dual-band absorption enhancement of graphene can be achieved for both the TE and TM polarizations. The results of cross-shaped graphene arrays can be generalized for designing more sophisticated metamaterials and related devices based on the patterned graphene structure.
